# Small RNAs from plants, bacteria and fungi within the order *Hypocreales* are ubiquitous in human plasma

**DOI:** 10.1186/1471-2164-15-933

**Published:** 2014-10-25

**Authors:** Meabh Beatty, Jasenka Guduric-Fuchs, Eoin Brown, Stephen Bridgett, Usha Chakravarthy, Ruth Esther Hogg, David Arthur Simpson

**Affiliations:** Centre for Experimental Medicine, Queen’s University Belfast, Belfast, Northern Ireland, UK

**Keywords:** Small RNAs, Fungi, Plasma, Microbiome, Metagenomics, Next generation sequencing, MicroRNA, Biomarker, Blood, Y RNA

## Abstract

**Background:**

The human microbiome plays a significant role in maintaining normal physiology. Changes in its composition have been associated with bowel disease, metabolic disorders and atherosclerosis. Sequences of microbial origin have been observed within small RNA sequencing data obtained from blood samples. The aim of this study was to characterise the microbiome from which these sequences are derived.

**Results:**

Abundant non-human small RNA sequences were identified in plasma and plasma exosomal samples. Assembly of these short sequences into longer contigs was the pivotal novel step in ascertaining their origin by BLAST searches. Most reads mapped to rRNA sequences. The taxonomic profiles of the microbes detected were very consistent between individuals but distinct from microbiomes reported at other sites. The majority of bacterial reads were from the phylum *Proteobacteria*, whilst for 5 of 6 individuals over 90% of the more abundant fungal reads were from the phylum *Ascomycota*; of these over 90% were from the order *Hypocreales*. Many contigs were from plants, presumably of dietary origin. In addition, extremely abundant small RNAs derived from human Y RNAs were detected.

**Conclusions:**

A characteristic profile of a subset of the human microbiome can be obtained by sequencing small RNAs present in the blood. The source and functions of these molecules remain to be determined, but the specific profiles are likely to reflect health status. The potential to provide biomarkers of diet and for the diagnosis and prognosis of human disease is immense.

**Electronic supplementary material:**

The online version of this article (doi:10.1186/1471-2164-15-933) contains supplementary material, which is available to authorized users.

## Background

It has been estimated that there are at least ten times more microbial cells associated with our bodies than there are human cells [1, 2]. Recent advances in high throughput, metagenomic sequencing approaches have facilitated identification of this diverse population of microbes at the genomic level. Characterisation of this microbiome, led by the Human Microbiome Project [3], has revealed that its composition varies widely between body sites and between individuals [2, 4–7].

The microbiome has a significant influence upon health. The majority of microbes are found in the gut and have essential roles in normal human physiology and immune responses [1, 8]. The composition of the gut microbiome is correlated with diet [9] and may be linked with the pathophysiology of bowel disorders [10, 11], obesity [12–14], atherosclerosis [15–17], diabetes [18], rheumatoid arthritis [19, 20] and neurodevelopmental disorders [21]. Inflammatory bowel conditions have been linked with the intestinal fungal community [4, 22].

Most metagenomic studies to date have involved isolation of DNA from external body sites or from the respiratory or digestive tracts, with fecal samples being the most commonly used source for investigation of the gut microbiome. Certain small RNAs are stable in the blood and in particular microRNAs have been widely studied as potential predictors of disease [23, 24]. However, we and others [25–27] have observed the existence of additional, exogenous small RNAs of potential microbial origin. Indeed, Wang *et al.* have documented the existence of RNA from bacteria and fungi in plasma and suggested that they may serve as signaling molecules or indicators of human health [25]. The origin of these small RNAs is unclear, but they are almost certainly derived from microbes inhabiting the gut or respiratory tract, rather than from viable microbes within the circulation. Nonetheless, it seems likely that the subset of the total human microbiome which contributes to these blood-borne small RNAs is linked with health status. The ability to reliably determine the composition of this microbiome from the sequences of the small RNAs present in a blood sample could form the basis of an extremely valuable diagnostic test.

The aim of this study was to construct a profile of the microbiome from which the exogenous small RNAs present in human plasma are derived. The merging of overlapping sequences to generate contigs facilitated identification of the origin of the short RNA sequences. The microbiome profiles generated were consistent across 6 individuals (3 from this study and 3 from publicly available data [28]). In addition to bacterial sequences, a large proportion of reads matched fungal sequences. To our surprise, the majority of these were assigned to the order *Hypocreales*. This work has further demonstrated the feasibility of generating a microbiome profile from small RNAs in plasma [25]. The ease of obtaining blood samples will facilitate analysis of this microbiome in a wide range of physiological and disease conditions. These findings also raise the intriguing questions of whether these exogenous RNAs have any functional implications and why sequences from one fungal order are so abundant.

## Results

**Figure 1:**
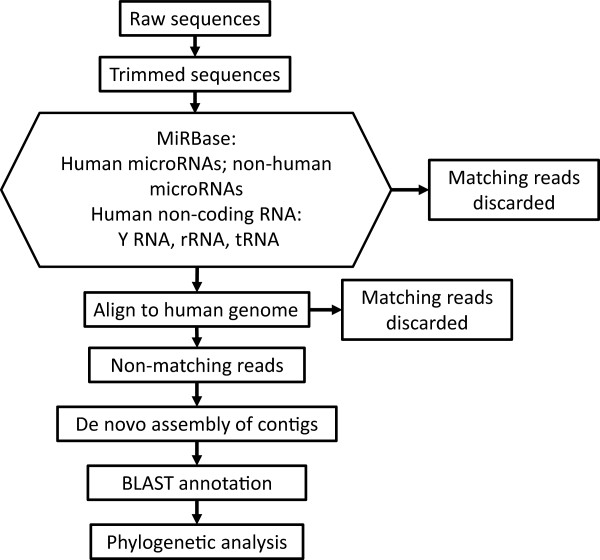
**Schema of the strategy for analysis of sequencing data.** Reads that did not align to human sequences or other known microRNAs were assembled into contigs. These were annotated by BLAST alignment to the NCBI nr database and phylogenetic analysis performed with the gi numbers of the top resulting hits.

RNA was extracted from three plasma samples and small RNA libraries prepared using an Illumina kit. Each library was sequenced on a MiSeq (Illumina). The unique reads and raw sequencing data have been deposited in Gene Expression Omnibus (GEO), accession number GSE52981. Sequencing data for three plasma exosomal small RNA libraries prepared with a kit from Bioo Scientific were downloaded from GEO [28]. For one of these samples data from libraries prepared with an NEB kit and an Illumina kit (as used in this study) were also available. The strategy for analysis of the sequencing data was to filter out reads derived from human genes, assemble the remaining reads into contigs, annotate these by alignment to known sequences and perform a phylogenetic classification (Figure 1).

**Figure 2:**
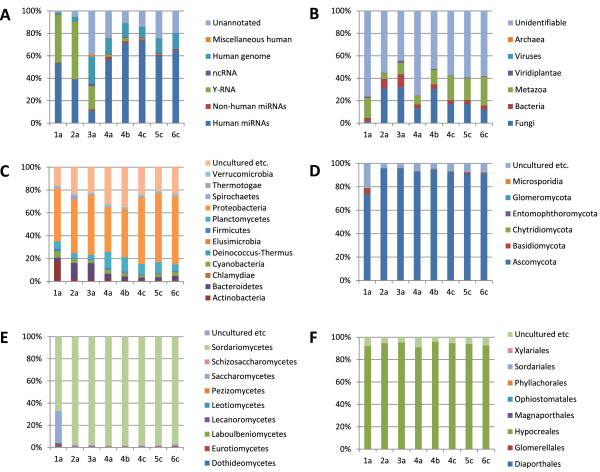
**Distribution of human reads by gene type and other reads by organism.** Each individual is represented by a number: 1–3 this study (whole plasma); 4–6 Huang et al. [28] (plasma exosomal RNAs). The library preparation method is indicated as follows: a = Illumina; b: NEB; c = Bioo Scientific). **(A)** 100% stacked columns illustrating the proportions of reads annotated to human genes, non-human microRNAs or unannotated. **(B)** The proportions of unannotated reads (from (a)) subsequently assigned to superkingdom or kingdom. **(C)** Bacterial reads assigned to Phyla (those comprising <0.5% in all samples are not illustrated). **(D)** The proportions of fungal reads by phyla. **(E)** The proportions of reads assigned to classes within the phylum *Ascomycetes*. **(F)** The proportions of reads assigned to orders within the class *Sordariomycetes*.

**Figure 3:**
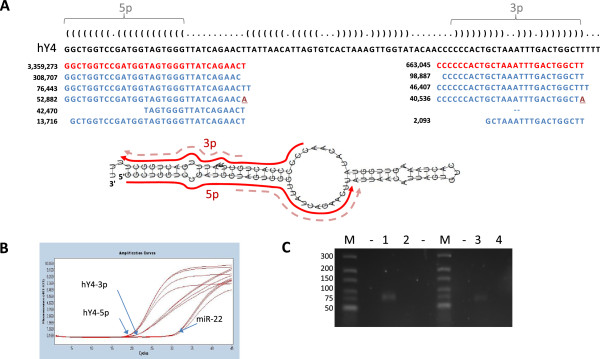
**Small RNAs derived from the non-coding hY4 RNA present in plasma. (A)** The predicted secondary structure of hY4 is shown in dot-bracket notation above the sequence and the reads mapping to the 5p and 3p arms indicated below (numbers refer to the reads detected in sample 1a). The positions of the most abundant 5p and 3p reads (and much less frequent short reads) are indicated by arrows adjacent to the hY4 structure. **(B)** Custom Taqman small RNA assays targeting the hY4-5p or 3p RNAs corresponding to the most abundant reads amplified products several threshold cycles before individual microRNAs (eg miR-22 in sample 1a). **(C)** RT-PCR with primers specific for the putative hY4 fragments and performed upon RNA that had been polyadenylated, amplified products with lengths consistent with the presence of the small RNA templates detected in the sequencing rather than full length hY4 RNA. A product of the predicted size (79 bp) was detected with the hY4 5p primer, whereas a longer product of 143 bp would have been amplified from full length hY4 RNA. M: Marker, sizes in bp; Lane 1, hY4 5p; Lane 2, No RT; Lane 3, hY4 3p; Lane 4, No RT.

The proportions of reads annotated to human genes are illustrated in Figure 2A (absolute numbers in Additional file 1). As expected, a large proportion of reads represented microRNAs, but remarkably, in the whole plasma samples prepared in this study, a similar proportion mapped to Y RNAs. Y RNAs are small cytoplasmic non-coding RNAs that can be cleaved to form smaller RNAs independently of the microRNA pathway [29]. The vast majority of reads (>99%) mapped to hY4, with small numbers to hY5, hY3 and hY1. A smaller but significant number of Y RNA sequences were present in the plasma exosome samples. In small RNA sequencing datasets from whole blood, which included cellular RNAs (GEO accession GSE46579), hy4-derived RNAs were present at levels comparable to an abundant microRNA [30]. The differences in Y RNA abundance observed between studies can be attributed to differences in sample collection (eg whole plasma or plasma exosomes) and library preparation, which result in differing distributions of small RNA read lengths (Additional file 2: Figure S1). The small RNAs detected corresponded to the 5p and 3p arms of the predicted secondary structure of hY4 (Figure 3A). Taqman small RNA RT-qPCR assays employ a stem-loop reverse transcription primer and are therefore expected to be specific for the target small RNA and not detect the full length precursor RNA. Therefore the low Cp values observed with the assays targeting the most abundant hY4 sequences from each arm both confirmed the presence of these small RNAs in plasma and suggested that they are indeed much more abundant than any individual microRNA (Figure 3B). To further confirm the presence of hY4 fragments, RNA was polyadenylated, reverse transcribed with an oligo-dT adaptor and PCR performed with primers specific for the putative hY4 fragments. The size of the product amplified using the 5p primer was consistent with presence of the small RNA template detected in the sequencing rather than full length hY4 RNA (Figure 3C).

A significant number of unannotated reads remained in all samples. The randomly cloned DNA sequences obtained in conventional metagenomic studies are typically assembled into contigs to enhance identification of homology with known genes. Although this strategy would not be applicable to discretely processed small RNAs, such as microRNAs, we reasoned that it could aid detection of longer RNAs which are processed to generate multiple small overlapping RNAs. All the unannotated reads were therefore pooled and assembled into 41542 contigs. For annotation purposes, the 5142 contigs with significant hits (E < 1×10^−3^) in a megablast search of the NCBI non-redundant database were assigned the identity of the top hit (lineages listed in Additional file 3). The unnannotated reads from each sample were realigned to these contigs and the proportions of reads mapping to different taxonomic categories calculated (Figure 2B-F). Most identifiable reads were assigned to Metazoa, Bacteria or Fungi. Although some metazoan reads could be derived from food [31], many are likely to be misassigned due to similarity with human sequences.

**Figure 4:**
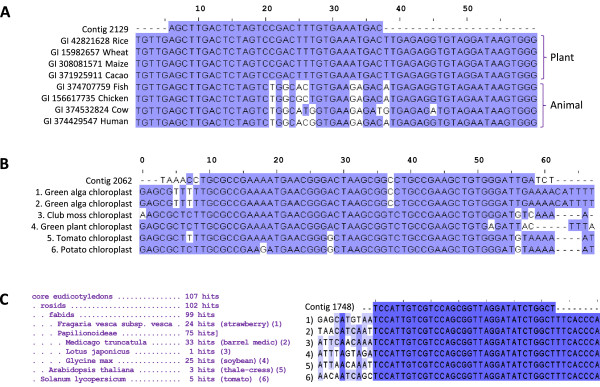
**Alignment of contigs with sequences that could potentially be derived from food.** Selected BLAST hits aligned using MAFFT and visualised with Jalview, coloured by BLOSUM62 score. **A)** Contig 2129 exhibits complete identity across the kingdom Viridiplantae 28S rRNA. Alignment with potential dietary plant and meat foodstuffs and the human rRNA gene. **B)** Contig 2062 is very similar to many chloroplast rRNA sequences and is shown aligned to several of the best hits and potential dietary sources. 1 - *Pseudendoclonium akinetum*: 2 - *Trichosarcina mucosa*: 3 - *Lycopodium clavatum*: 4 - *Zygnema*: 5 - *Solanum Lycopersicum*: 6 - *Solanum tuberosum*: **C)** All the lineages to which Contig 1748 has a perfect match, including many potential food sources. Representative sequences from each species are aligned and coloured by percentage identity (1: *Fragaria vesca*, 2: *Medicago truncatula*, 3: *Lotus japonicus*, 4: *Glycine max*, 5: *Arabidopsis thaliana*, 6: *Solanum lycopersicum*, NB: A 30 bp insertion present in *Glycine max* immediately 5 prime of the contig 1748 sequence is omitted to facilitate visualisation). Full lineage of core eudicotyledons is [root; cellular organisms; Eukaryota; Viridiplantae; Streptophyta; Streptophytina; Embryophyta; Tracheophyta; Euphyllophyta; Spermatophyta; Magnoliophyta; eudicotyledons].

A small percentage of contigs matched plant sequences, but due to the conservation of rRNA across the kingdom *Viridiplantae*, the top blast hits did not reliably identify their source, but rather reflected the composition of the database (a preponderance of algal sequences was observed). However, in most instances the sequences were sufficiently divergent from human rRNA to support the notion that they are derived from dietary plant material (Figure 4).

**Figure 5:**
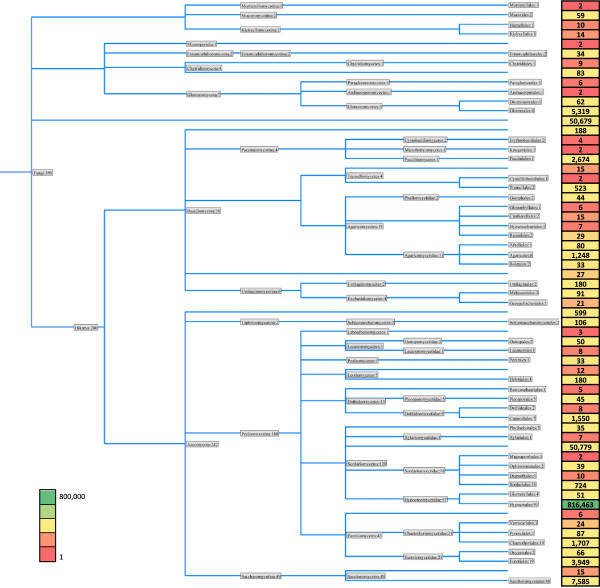
**Order-level phylogenetic profile of fungal small RNAs.** The tree illustrates the taxonomic composition of the contigs derived from small RNAs isolated from the plasma samples of six individuals. All orders within the kingdom *Fungi* which have matching sequences are illustrated. The numbers of contigs assigned to each taxonomic group are indicated within the tree. The numbers on the right are the total number of reads assigned to each order; the order *Hypocreales*, highlighted in green, is the most abundant.

The phylogenetic profile of the bacterial microbiome was remarkably similar between individuals (Figure 2C), with *Proteobacteria* being the most abundant phylum*.* This is consistent with an origin in the gut. The number of reads matching fungal sequences was higher than expected and of these, more than 90% in 5 of 6 individuals were from the phylum *Ascomycetes* (Figure 2D). Remarkably, it was possible to further define the origin of almost all these reads to within the class *Sordariomycetes* and order *Hypocreales* (Figure 2E-F)*.* The predominance of sequences from the *Hypocreales* is illustrated when the numbers of reads mapping to each fungal order are placed on a phylogenetic tree comprising all orders with at least one matching contig (Figure 5).

**Figure 6:**
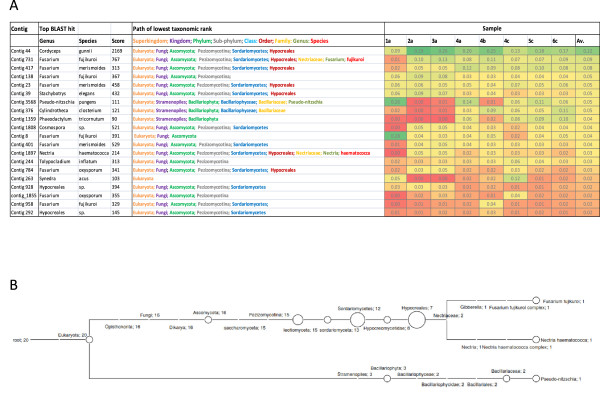
**Taxonomic profile and relative expression between individuals of abundant contigs. (A)** The top 20 contigs ranked according to the total number of reads aligned to them from all samples. All the contigs matched rRNA and the top BLAST hit is shown. The lowest common taxonomic rank was assigned by analysis of the BLAST hits with scores within 5% of the top hit. The proportion of reads mapping to each contig in individuals and overall is indicated. **(B)** Phylogenetic tree of the top 20 contigs generated with MEGAN. The number of contigs assigned at each node is indicated.

For the 20 exogenous contigs represented by the most reads, the top 5% of BLAST hits (min score 50) were analysed with the MEGAN taxonomic classification tool [32, 33]. They all mapped to rRNA, 16 of the 20 to fungal sequences, with the lowest common taxonomic rank for 5 of the top 6 being the fungal order *Hypocreales* or lower (Figure 6). The relative abundances of contigs across the samples were very consistent. Contig 44, which mapped to *Hypocreales* rRNA, was the most abundant in 5 of the 6 individuals. Notably 9 of the top BLAST hits for the 20 contigs were to the genus *Fusarium*. The mycoprotein Quorn is derived from *Fusarium venenatum* [34]. Although it is intriguing to speculate that the sequences we observe are derived from Quorn, it seems unlikely that all 6 subjects would have had this in their diet. In addition, although several contigs align very closely with published *F. venenatum* rRNA sequences, they match even more closely to other species (Additional file 4: Figure S2).

**Figure 7:**
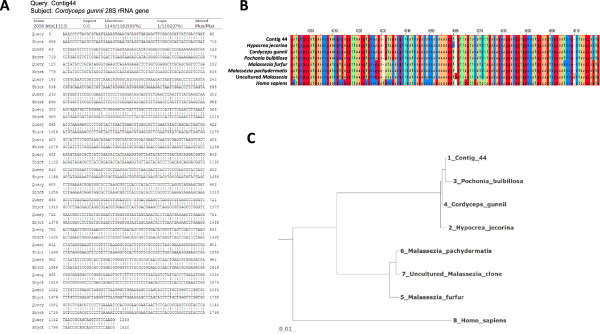
**Alignment of contig 44 to rRNA sequences. (A)** BLAST alignment of contig 44 with *Cordyceps gunnii* 28S ribosomal RNA gene. **(B)** Section of multiple alignment between contig 44 and rRNA sequences from exemplar species in the orders *Hypocreales* or *Malasseziales* and human rRNA. **(C)** Phylogram illustrating the divergence between *Hypocreales*/contig 44, *Malasseziales* and human rRNA sequences.

The contigs assigned to *Hypocreales* are extremely similar to the published sequences. For example, contig 44 has a similarity of 98.6% identity over 1162 nucleotides to *Hypocreales Cordycipitaceae Cordyceps gunnii* 28S ribosomal RNA (Figure 7A). This contig can also be aligned, with lower similarity, to rRNA from many other species. A region of contig 44 across which many orthologous sequences were available was selected and a multiple alignment made (Figure 7B). The phylogram derived from this alignment illustrates that contig 44 is considerably more similar to sequences from several species within *Hypocreales* than to those within *Malasseziales* and even more dissimilar to the human rRNA sequence (Figure 7C). Contigs generated from analysis of samples from the study by Wang *et al.* [25] were also similar to fungal sequences and indeed some were identical to contig 44 for >700 bp (Additional file 5: Figure S3).

**Figure 8:**
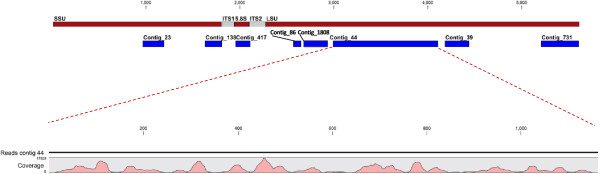
**Distribution of reads along** ***Hypocreales*** **rRNA gene.** The positions of the most abundant contigs along the rDNA are indicated at the top of the figure. The read coverage for contig 44 is shown. Abbreviations: SSU: Small subunit; LSU: Large subunit; ITS: Internal Transcribed Sequence.

All the most abundant contigs fall within the mature rRNA regions but the distribution of detected reads is very uneven (Figure 8). Although the variation in coverage could be partially due to experimental bias (ie differential cloning efficiency of sequences [35]) it is also likely to reflect *in vivo* abundances.

## Discussion

Highly expressed small RNAs derived from Y RNAs hY1 and hY3 have been reported in tumours and high expression in serum suggested by RT-PCR [36]. We also observed a small number of sequences matching hY1 and hy3, but the presence of extremely abundant hY4 fragments, confirmed by RT-qPCR, was unexpected. Our ability to detect Y RNA fragments as such a large proportion of total small RNAs in this study may relate to practical details of the library preparation protocol employed, particularly the size range selected. Y RNAs form part of the RoRNP, which also contains the proteins Ro60 and La, but their function is poorly understood [37]. They are required for chromosomal replication [38] and are overexpressed in tumours [39]. It has been demonstrated that double-stranded RNA oligonucleotides comprising the stem of the Y RNA are sufficient to reconstitute DNA replication *in vitro* [40]. Y RNAs are rapidly degraded during apoptosis to generate fragments similar in size to those observed in this study [41]. Although it has been suggested that small RNAs derived from Y RNAs may act analogously to microRNAs, the formation of Y3 and Y5 RNA fragments has been shown to be Dicer independent [29]. Given the abundance of the hY4 fragments in plasma, it is an intriguing possibility that they may have some, as yet unknown function.

The detection of microbial sequences in plasma supports previous reports of circulating enterobacterial transcripts [27] and the most detailed study of these sequences to date by Wang *et al.* [25], who performed extensive control experiments to rule out potential sources of contamination. However, the possibility that observations of exogenous RNAs result from contamination remains a serious concern [42]. Spurious detection of such sequences could arise due to contamination during sample handling, library preparation or sequencing or result from errors in data analysis. It is difficult to envisage how contamination with identical sequences could occur in studies undertaken in diverse locations by independent investigators, ie as detected in this study and by Huang *et al.* [28] and Wang *et al.* [25] (Additional file 5: Figure S3). In addition, analysis of data from the sequence runs prior to those reported in this study confirmed that they were not the source of contamination. In this study reads were assembled to try to improve mapping accuracy and reduce the computational requirements for database searching. The observation of similar mapping results without assembly of the sequence reads (Additional file 6: Figure S4) supports the proposed phylogenetic origins.

The taxonomic breakdown of the originating organisms achieved with our contig-based strategy is in broad agreement with that reported by Wang *et al.*; *Proteobacteria* were the most abundant bacterial phylum in both studies, with *Bacteroidetes* also commonly detected, whilst *Ascomycota* was the most abundant phylum of Fungi in both studies. However, our data suggest an even greater predominance of *Ascomycota* and we can assign many of these reads down to the level of Order (*Hypocreales*). Whilst members of this order have occasionally been reported as opportune pathogens in immunocompromised patients [43], they are more commonly plant or insect parasites [44], while *Hypocrea jecorina* is a widely used source of cellulases [45]. It is remarkable that the vast majority of fungal reads should be derived from a small number of closely related species or potentially even a single species. From where do all these sequences originate?

The composition of both the fungal and bacterial plasma microbiome detected suggests that the sequences do not result from contamination from the skin microbiome during collection of blood samples. Whilst the human skin microbiome varies widely, it is dominated by the bacterial phylum *Actinobacteria* (and to a lesser degree *Firmicutes* and *Proteobacteria*) [1, 5] and the fungal genus *Malassezia* of the *Basidiomycota* phylum [6]. Reads from *Actinobacteria* comprised an average of 1.5% percent of bacterial reads in 5 of 6 samples and only 17.6% in the remaining sample. *Firmicutes* averaged 1% percent across all samples, although *Proteobacteria* were the most abundant (50%). With regard to fungi, only 3 contigs (91 reads) were assigned to *Malassezia*. It seems unlikely that contamination during sample processing could result in such similar microbiome profiles in three independent plasma small RNA datasets and across multiple library preparation methods.

Small RNA sequences have been reported to enter the circulation from the gastrointestinal tract [31] and pharmacological preparations of small interfering RNAs (siRNAs) have been demonstrated to cross the gut wall following oral administration [46–48]. The gut therefore seems the most likely origin for microbial plasma small RNAs. The human gut, in contrast to skin, is predominantly colonised by the bacterial phyla *Bacteroidetes* and Firmicutes [1, 5], and by the fungal phylum *Ascomycota* [4]. It is therefore conceivable that the gut is the source, but one would not expect the observed predominance of sequences from *Hypocreales.* Perhaps the niche occupied by these species within the gut predisposes them to uptake into the circulation. The respiratory tract is another potential source and indeed *Fusarium* is one of the four most common pathogenic fungi detected, along with *Candida*, *Aspergillus* and *Cryptococcus* [49]. Although some microRNAs may be absorbed from the gut unshielded to survive exposed in the circulation for several hours [31, 50], many are protected from degradation by association with lipids and proteins [51, 52] and there is some evidence that the exogenous RNAs may be similarly protected [25]. Indeed rRNA fragments have been shown to enter argonaute protein complexes [53]. Differential stability could contribute to over-representation of certain sequences.

In addition to RNAs of microbial origin, some sequences potentially derived from foodstuffs were detected. Notably the greatest proportion of reads matching plant sequences were found in sample 3, which was obtained from the one individual who reported following a vegetarian diet. Although it has been reported that plant microRNAs (xenomiRs) are not reliably detected in plasma after ingestion [54, 55] the possibility of genetic material from food entering the circulation is supported by the detection of plant chloroplast DNA in the blood of cows [56]. The unequivocal assignment of significant numbers of circulating small RNAs to plant rRNA in this study raises the exciting possibility that it may be possible to quantify diet from a simple blood test.

Great care must be taken when comparing between studies because differences in sample collection and library preparation can have profound effects upon the small RNA profiles observed and the proportion of reads mapping to Y RNAs or exogenous small RNAs. Nonetheless, the detection of these same small RNAs in diverse studies confirms that they are a common feature of the circulation.

## Conclusion

Abundant fragments derived from the non-coding hY4 RNA, but of unknown function, have been detected in human plasma. RNAs from a diverse range of microbes are also present, but the majority of fungal sequences are from species in the Order *Hypocreales*. This raises questions about how these exogenous RNAs reach the circulation, whether they are functional and why specific fungi are so highly represented. This work has demonstrated the feasibility of determining the microbiome that contributes small RNAs to the blood. The profile of microbial sequences detected is almost certainly influenced by the composition of the wider microbiome, particularly in the gut. Given the integral role of the human microbiome in normal health and pathology, it seems likely that knowledge of the plasma microbiome will be soon prove to be of clinical importance.

## Methods

### Sample collection and RNA extraction

Three healthy individuals aged 20–40 years were recruited from Belfast, N. Ireland, UK: male, Caucasian (sample 1); female, Caucasian (sample 2); and male, Indian (sample 3). All participants completed a food-frequency questionnaire which included questions on any special dietary requirements. A blood sample was taken in EDTA-treated tubes and plasma was separated immediately by centrifugation for 10 minutes at 1,000 *g* and subsequently at 10,000 *g* for 10 minutes prior to RNA extraction using a miRNeasy kit (Qiagen, Crawley, UK). RNA purity and quantity were determined using a Nanodrop spectrophotometer (Thermo Scientific) and Qubit fluorimeter (Life Technologies). RNA integrity was assessed using RNA 2000 and small RNA chips on a Bioanalyzer (Agilent).

### Ethics and consent

This study was conducted according to the guidelines laid down in the Declaration of Helsinki and all procedures involving human participants/patients were approved by the Research Ethics Committee of the School of Medicine and Dentistry, Queen’s University Belfast (Ref:11/05v3). Written informed consent was obtained from all participants.

### Deep sequencing

Small RNA libraries were prepared using a Truseq small RNA sample prep kit (Illumina) following the manufacturer’s protocol. This included size selection using a 6% PAGE Gel; the region between the custom Illumina markers was excised, corresponding to insert sizes of approximately 20–35 nucleotides. Cluster generation and sequencing with 40 nucleotide reads on a MiSeq was performed at the Trinity Genome Sequencing Laboratory, Dublin [57].

### Data analysis

Sequencing data were analyzed using Genomics workbench software v5.5.1 (CLCbio, Aarhus, Denmark). After removal of adapter sequences, reads >15 bp and with at least 2 copies were aligned, allowing 2 mismatches, to miRBase (Release 19), a database of human non-coding RNA downloaded from Ensembl using Biomart [58] and the human genome (hg19). The remaining unannotated reads were pooled and assembled into contigs using the de novo assembly algorithm of Genomics workbench. Reads from each individual sample were then mapped back to the contigs. For subsequent phylogenetic analyses the putative origins of contig sequences were assigned using the sequence identifier (gi) numbers of the top hits determined by megablast [59, 60] (available online [61]) against the NCBI non-redundant database (E-value <0.001). Lists of gi numbers were uploaded to the metagenomic analysis tools [62] available through the Galaxy platform [63, 64], specifically to ‘Fetch taxonomic representation’, ‘Summarize taxonomy’, ‘draw phylogeny’ and ‘Find lowest diagnostic rank’. Microsoft Access databases were used to integrate datasets. Taxonomic classification of the top 5% of BLAST hits was performed using the MEtaGenome ANalyzer (MEGAN) analysis tool [32, 33]. The lowest common ancestor was assigned following manual removal of individual hits with obviously incorrect taxonomic classifications (ie matching the query and top blast hits but not other sequences from their alleged species). Optimal RNA secondary structures were predicted using the Vienna RNAfold webserver [65, 66]. Additional multiple sequence alignments were performed using the Multiple Alignment using Fast Fourier Transform (MAFFT) program [67] available online [68] or Clustal Omega [69, 70], available through the EBI server [71]. Multiple alignments were visualised with Jalview [72] and phylograms with Archaeopteryx [73]. Custom Perl scripts were used for manipulating sequence files.

### RT-PCR

Y-RNA custom small RNA Taqman assays (Life Technologies) were designed to target the following sequences: HY4_5p; GGCUGGUCCGAUGGUAGUGGGUUAUCAGAACU and HY4_3p; CCCCCCACUGCUAAAUUUGACUGGCUU . Taqman reverse transcription and PCR were performed according to the manufacturer’s instructions on a LightCycler480 platform (Roche).

For detection of Y-RNA fragments, RNA was polyadenylated using *E. coli* Poly(A) Polymerase I (Ambion) and reverse transcribed using Super Script III reverse transcriptase (Life Technologies) and an oligo-dt adaptor: GCGAGCACAGAATTAATACGACTCACTATAGGTTTTTTTTTTTTVN. PCR was performed using the common reverse primer GCGAGCACAGAATTAATACGAC and either an HY4_5p primer: GGCTGGTCCGATGGTAGT or HY4_3p primer: CCCCCCACTGCTAAAATTTGA. 35 cycles of PCR were performed with the following conditions 94°C 30 sec; 56°C 30 sec; 72°C 1 minute using Hotstar Taq DNA polymerase (Qiagen).

### Availability of supporting data

The data sets supporting the results of this article are available in the Gene Expression Omnibus (GEO) repository [74]. The sequencing data generated in this study has accession number GSE52981 ( https://www.ncbi.nlm.nih.gov/geo/query/acc.cgi?acc=GSE52981) and the publicly available plasma small RNA sequencing data [28] analysed has accession number GSE45722.

## Electronic supplementary material

Additional file 1: **Excel spreadsheet.** Number of reads from each sample annotated by gene type or phylogenetic category (XLSX 19 KB)

Additional file 2: Figure S1: Distribution of read lengths in a range of sequencing libraries prepared from blood. The percentages of reads of each length are shown for libraries prepared from plasma, exosomes isolated from plasma or whole blood, including cells. Both the source material and library preparation protocol (eg size selection) influence the insert sizes observed. References: Huang *et al.* [28]; Wang *et al.* [25]; Leidinger *et al.* [30]. (PDF 172 KB)

Additional file 3: **Excel spreadsheet.** Full lineages of the top blast hits of all contigs. (XLSX 660 KB)

Additional file 4: Figure S2: Multiple alignment of Contig_1808 with fungal rRNAs. Contig 1808 is aligned with the five most similar sequences in the NCBI nr database and the rRNA sequence of *Fusarium venenatum*. (PDF 283 KB)

Additional file 5: Figure S3: Analysis of plasma sequence data from Wang *et al.* [25]. (a) Taxonomic composition of the contigs derived from small RNAs isolated from a normal plasma sample (ERR248695), determined from BLAST searches using MEGAN. (b) Alignment of one contig derived from sample ERR248695 from the study by Wang et al. [25] with contig 44 from this study, demonstrating total identity. (PDF 145 KB)

Additional file 6: Figure S4: Phylogenetic profiles predicted from individual reads or contigs. A random subset of the reads that were unannotated to human databases was generated from Sample 3A. These were input either directly or after assembly into contigs to BLAST searches of the nt database. Similarities with fungal sequences are a key feature detected by both approaches. Using this subset of sequences no contigs with potential bacterial origin were detected, probably reflecting the relatively low abundance of putative bacterial reads in this sample in comparison to fungal reads (see Figure 2D). (a) Phylogenetic profile predicted using MEGAN to interpret BLAST searches using contigs assembled from the reads. The number of hits at each node is indicated. (b) Phylogenetic profile predicted from BLAST searches of individual reads. The similarity between the trees suggests that mapping of assembled reads is broadly consistent to the results with individual reads. (PDF 150 KB)
