# Supplementary figures and images for: Small RNAs from plants, bacteria and fungi within the order Hypocreales are ubiquitous in human plasma
Source: BMC Genomics. 2014 Oct 25;15(1):933. doi: 10.1186/1471-2164-15-933 (PMC4230795; doi:10.1186/1471-2164-15-933)

Suppl Fig 1

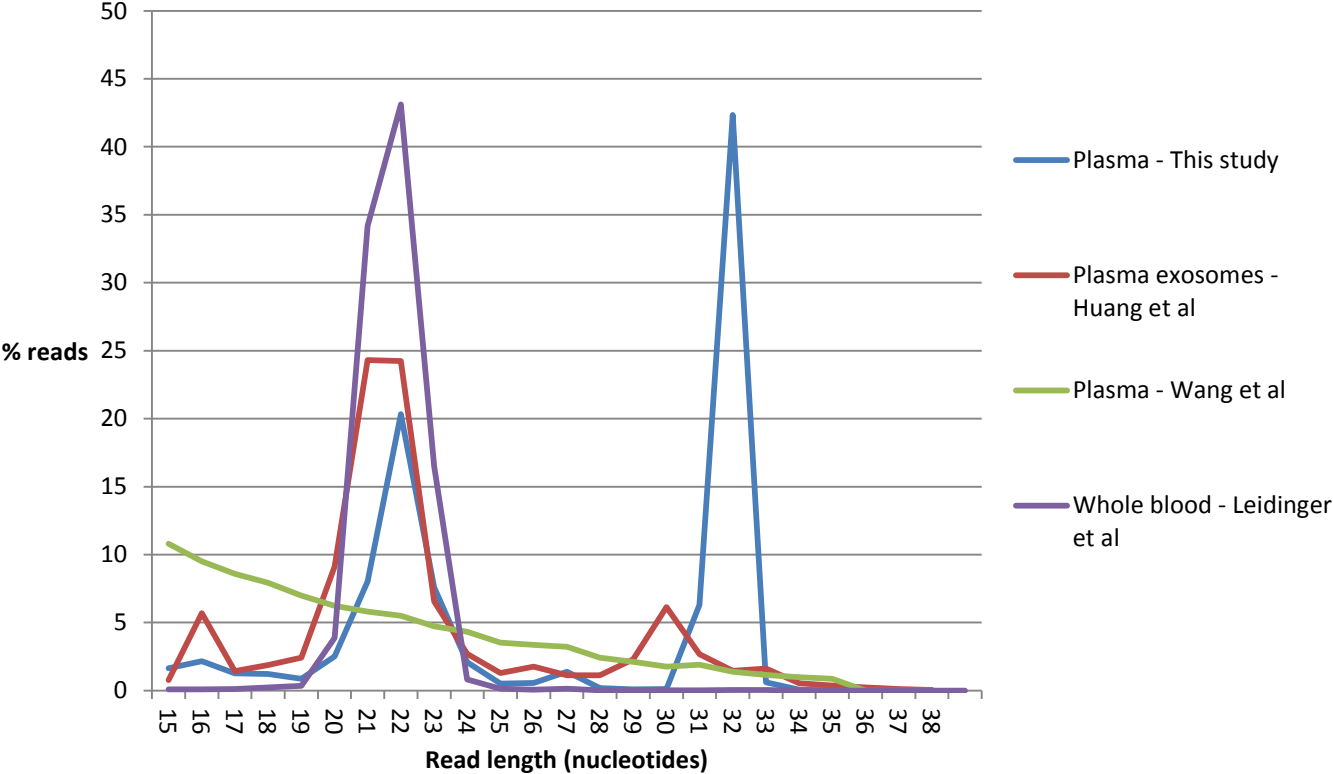

Supplement: Supplementary file 2 — Additional file 2: Figure S1: Distribution of read lengths in a range of sequencing libraries prepared from blood. The percentages of reads of each length are shown for libraries prepared from plasma, exosomes isolated from plasma or whole blood, including cells. Both the source material and library preparation protocol (eg size selection) influence the insert sizes observed. References: Huang et al. [28]; Wang et al. [25]; Leidinger et al. [30]. (PDF 172 KB) [file 12864_2014_6643_MOESM2_ESM.pdf]

Suppl Fig 4

A

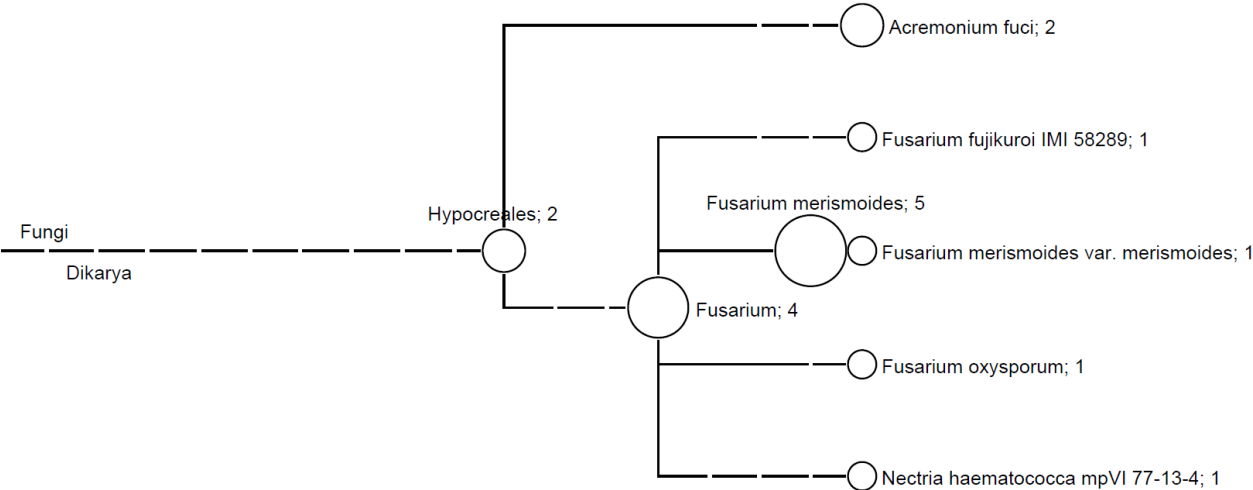

B

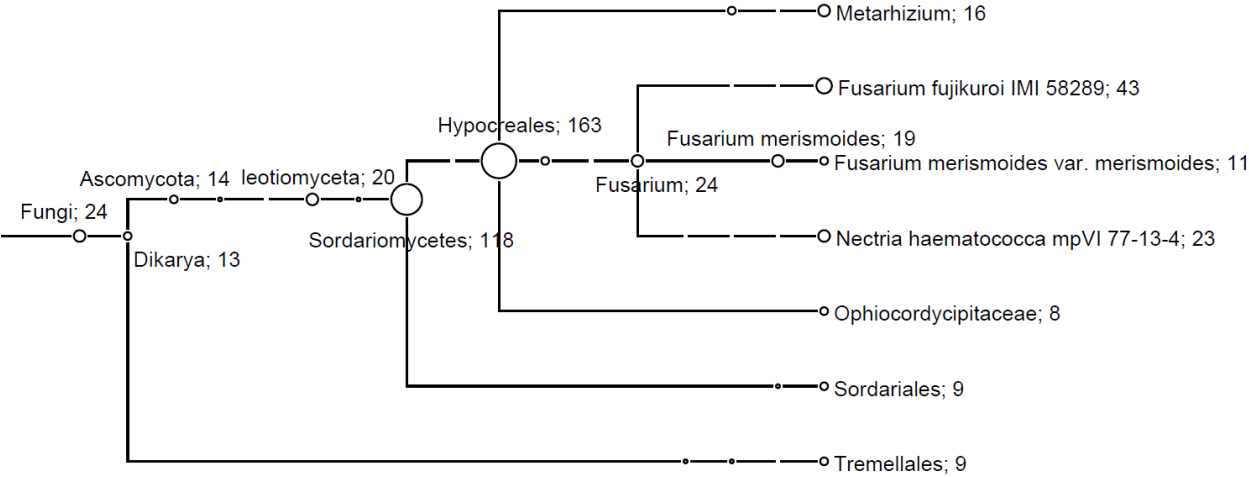

Supplement: Supplementary file 6 — Additional file 6: Figure S4: Phylogenetic profiles predicted from individual reads or contigs. A random subset of the reads that were unannotated to human databases was generated from Sample 3A. These were input either directly or after assembly into contigs to BLAST searches of the nt database. Similarities with fungal sequences are a key feature detected by both approaches. Using this subset of sequences no contigs with potential bacterial origin were detected, probably reflecting the relatively low abundance of putative bacterial reads in this sample in comparison to fungal reads (see Figure 2D). (a) Phylogenetic profile predicted using MEGAN to interpret BLAST searches using contigs assembled from the reads. The number of hits at each node is indicated. (b) Phylogenetic profile predicted from BLAST searches of individual reads. The similarity between the trees suggests that mapping of assembled reads is broadly consistent to the results with individual reads. (PDF 150 KB) [file 12864_2014_6643_MOESM6_ESM.pdf]
