# Supplementary material for: Small RNAs from plants, bacteria and fungi within the order Hypocreales are ubiquitous in human plasma
Source: BMC Genomics. 2014 Oct 25;15(1):933. doi: 10.1186/1471-2164-15-933 (PMC4230795; doi:10.1186/1471-2164-15-933)
Supplement: Supplementary file 4 — Additional file 4: Figure S2: Multiple alignment of Contig_1808 with fungal rRNAs. Contig 1808 is aligned with the five most similar sequences in the NCBI nr database and the rRNA sequence of Fusarium venenatum. (PDF 283 KB) [file 12864_2014_6643_MOESM4_ESM.pdf]

Suppl Fig 2

```
Contig_1808                          CGCTGGTGCACTTGGCCGGCCCAGGCCAGCATCAGTTCGGTGTGGGGGATAAAGGCGGCGGGAATGTGGCTCCTC--CGGGAGTGTTATAGCCCACCGT
Cosmospora sp. GI:260515147          CGCTGGTGCACTTGGCCGGCCCAGGCCAGCATCAGTTCGGTGTGGGGGATAAAGGCGGCGGGAATGTGGCTCCTC--CGGGAGTGTTATAGCCCACCGT
Roselliniella atlantica GI:260600585 CGCTGGTGCACTTGGCCGGCCCAGGCCAGCATCAGTTCGGTGTGGGGGATAAAGGCGGCGGGAATGTGGCTCCTC--CGGGAGTGTTATAGCCCACCGT
Melanospora sp. GI:227215079         CGCTGGTGCACTTGGCCGGCCCAGGCCAGCATCAGTTCGGTGTGGGGGATAAAGGCGGCGGGAATGTGGCTCCTC--CGGGAGTGTTATAGCCCACCGT
Uncultured fungus GI:315190306       CGCTGGTGCACTTGGCCGGCCCAGGCCAGCATCAGTTCGGCGTGGGGGATAAAGGCGGCGGGAATGTGGCTCCTC--CGGGAGTGTTATAGCCCACCGT
Roselliniella sp. GI:260600586       CGCTGGTGCACTTGGCCGGCCCAGGCCAGCATCATTTCGGTGTGGGGGATAAAGGCGGCGGGAATGTGGCTCCTC--CGGGAGTGTTATAGCCCACCGT
Fusarium merismoides GI:218455080    CGCTGGTGCACTTGGCCGGCCCAGGCCAGCATCAGTTCGGCGTGGGGGATAAAGGCGGCGGGAATGTGGCTCCTC--CGGGAGTGTTATAGCCCACCGT
Fusarium venenatum GI:2957213        CCCCAGTGCACTTTTCCAGTCCAGGCCAGCATCAGTTTTCGCCGGGGGATAAAGACTTCGGGAATGTGGCTCCTCTCGGGGAGTGTTATAGCCCGTTGT
```
