# Supplementary material for: Small RNAs from plants, bacteria and fungi within the order Hypocreales are ubiquitous in human plasma
Source: BMC Genomics. 2014 Oct 25;15(1):933. doi: 10.1186/1471-2164-15-933 (PMC4230795; doi:10.1186/1471-2164-15-933)
Supplement: Supplementary file 5 — Additional file 5: Figure S3: Analysis of plasma sequence data from Wang et al. [25]. (a) Taxonomic composition of the contigs derived from small RNAs isolated from a normal plasma sample (ERR248695), determined from BLAST searches using MEGAN. (b) Alignment of one contig derived from sample ERR248695 from the study by Wang et al. [25] with contig 44 from this study, demonstrating total identity. (PDF 145 KB) [file 12864_2014_6643_MOESM5_ESM.pdf]

Suppl Fig 3

A

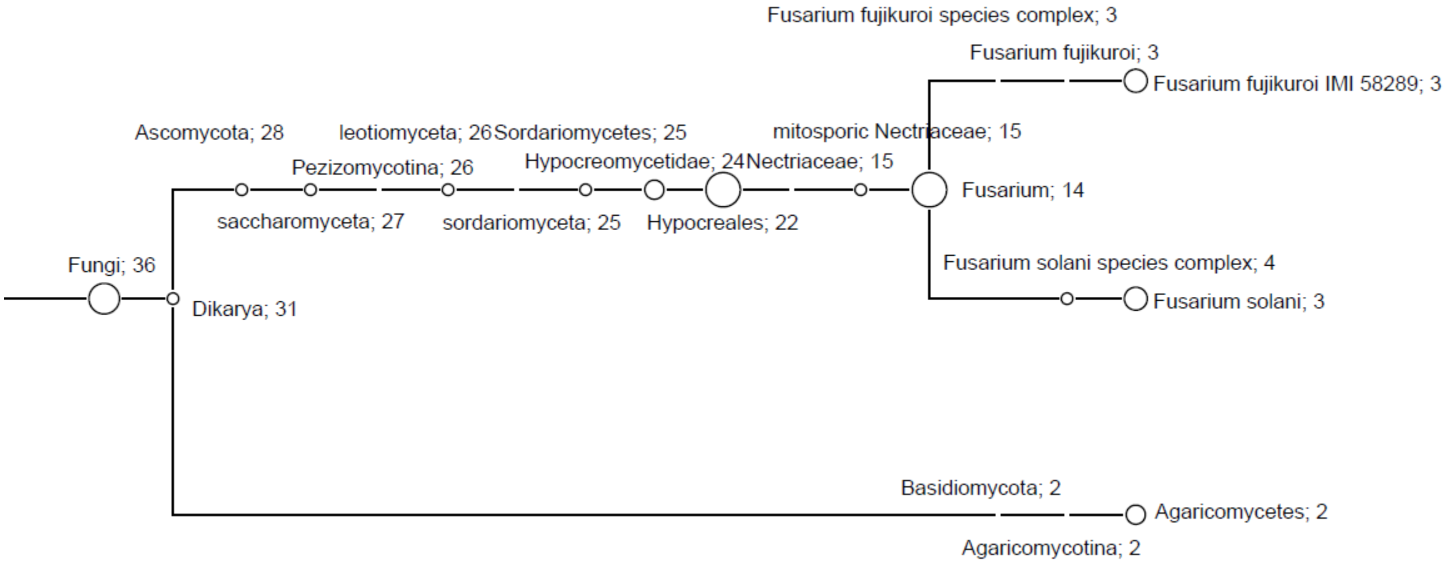

B

```
Score            Expect          Identities       Gaps            Strand
1334 bits(722)   0.0             722/722(100%)    0/722(0%)       Plus/Plus

ERR248695 213  GGGGCGAAAGACTAATCGAACCTTCTAGTAGCTGGTTTCCGCCGAAGTTTCCCTCAGGATAGCAGTGTTGAACTCAGTTTTATGAGGTAAAGCGAATGATTAGGGACTCGGGGGCGCTAT  332
               ||||||||||||||||||||||||||||||||||||||||||||||||||||||||||||||||||||||||||||||||||||||||||||||||||||||||||||||||||||||||
Contig_44    1  GGGGCGAAAGACTAATCGAACCTTCTAGTAGCTGGTTTCCGCCGAAGTTTCCCTCAGGATAGCAGTGTTGAACTCAGTTTTATGAGGTAAAGCGAATGATTAGGGACTCGGGGGCGCTAT  120

ERR248695 333  TTAGCCTTCATCCATTCTCAAACTTTAAATATGTAAGAAGCCCTTGTTACTTAATTGAACGTGGGCATTCGAATGTATCAACACTAGTGGGCCATTTTTGGTAAGCAGAACTGGCGATGC  452
               ||||||||||||||||||||||||||||||||||||||||||||||||||||||||||||||||||||||||||||||||||||||||||||||||||||||||||||||||||||||||
Contig_44 121  TTAGCCTTCATCCATTCTCAAACTTTAAATATGTAAGAAGCCCTTGTTACTTAATTGAACGTGGGCATTCGAATGTATCAACACTAGTGGGCCATTTTTGGTAAGCAGAACTGGCGATGC  240

ERR248695 453  GGGATGAACCGAACGCGAGGTTAAGGTGCCAGAGTAGACGCTCATCAGACACCACAAAAGGTGTTAGTACATCTTGACAGCAGGACGGTGGCCATGGAAGTCGGAATCCGCTAAGGACTG  572
               ||||||||||||||||||||||||||||||||||||||||||||||||||||||||||||||||||||||||||||||||||||||||||||||||||||||||||||||||||||||||
Contig_44 241  GGGATGAACCGAACGCGAGGTTAAGGTGCCAGAGTAGACGCTCATCAGACACCACAAAAGGTGTTAGTACATCTTGACAGCAGGACGGTGGCCATGGAAGTCGGAATCCGCTAAGGACTG  360

ERR248695 573  TGTAACAACTCACCTGCCGAATGTACTAGCCCTGAAAATGGATGGCGCTCAAGCGTCTCACCCATACCTCGCCCTCAGGGTAGAAACGATGCCCTGAGGAGTAGGCGGACGTGGAGGTCG  692
               ||||||||||||||||||||||||||||||||||||||||||||||||||||||||||||||||||||||||||||||||||||||||||||||||||||||||||||||||||||||||
Contig_44 361  TGTAACAACTCACCTGCCGAATGTACTAGCCCTGAAAATGGATGGCGCTCAAGCGTCTCACCCATACCTCGCCCTCAGGGTAGAAACGATGCCCTGAGGAGTAGGCGGACGTGGAGGTCG  480

ERR248695 693  TGACGAAGCCTAGGGCGTGAGCCCGGGTCGAACGGCCTCTAGTGCAGATCTTGGTGGTAGTAGCAAATACTTCAATGAGAACTTGAAGGACCGAAGTGGGGAAAGGTTCCATGTGAACAG  812
               ||||||||||||||||||||||||||||||||||||||||||||||||||||||||||||||||||||||||||||||||||||||||||||||||||||||||||||||||||||||||
Contig_44 481  TGACGAAGCCTAGGGCGTGAGCCCGGGTCGAACGGCCTCTAGTGCAGATCTTGGTGGTAGTAGCAAATACTTCAATGAGAACTTGAAGGACCGAAGTGGGGAAAGGTTCCATGTGAACAG  600

ERR248695 813  CGGTTGGACATGGGTTAGTCGATCCTAAGCTATAGGGAAGTTCCGTTTCAAAGGCGCACTTGTGCGCCGTCTAGCGAAAGGGGAGCCGGTCAATATTCCGGCACCTGGATGTGGGTTTTG  932
               ||||||||||||||||||||||||||||||||||||||||||||||||||||||||||||||||||||||||||||||||||||||||||||||||||||||||||||||||||||||||
Contig_44 601  CGGTTGGACATGGGTTAGTCGATCCTAAGCTATAGGGAAGTTCCGTTTCAAAGGCGCACTTGTGCGCCGTCTAGCGAAAGGGGAGCCGGTCAATATTCCGGCACCTGGATGTGGGTTTTG  720
```
